# Socio-ecological factors of health literacy and physical activity among middle-aged and older Chinese adults

**DOI:** 10.3389/fpubh.2026.1749276

**Published:** 2026-02-19

**Authors:** Li Huang, Yanxia Zhao, Haodong Tian, Haowei Liu, Yunfei Tao, Yuping Zhu, Mingyue Yin, Xing Zhang, Hansen Li

**Affiliations:** 1 Faculty of Psychology, Southwest University, Chongqing, China; 2 College of Physical Education, Chongqing University, Chongqing, China; 3 College of Physical Education, Southwest University, Chongqing, China; 4 School of Sport Training, Chengdu Sport University, Chengdu, China; 5 School of Athletic Performance, Shanghai University of Sport, Shanghai, China; 6 Department of Physical Education and Sport, Faculty of Sport Sciences, University of Granada, Granada, Spain; 7 School of Physical Education, Sichuan Agricultural University, Ya’an, China

**Keywords:** aging, older adults, public health, socio-demographic factors, socioeconomic status

## Abstract

**Background:**

In rapidly aging China, physical activity (PA) and health literacy (HL) are increasingly recognized as important components of healthy aging.

**Methods:**

A cross-sectional online survey was conducted among Chinese adults aged ≥40 years between July and September 2023 (*N* = 508). PA was assessed using the Physical Activity Rating Scale-3, and HL was measured using the Chinese version of the 12-item Health Literacy Scale Short-Form. Potential correlates spanning individual, behavioral, interpersonal, and community domains were examined using LASSO regression. The robustness of selected correlates was evaluated through bootstrap resampling and repeated random cross-validation.

**Results:**

Participants were predominantly urban-dwelling and highly educated, with most aged 40–54 years. For PA, regular exercise and sleep duration were identified as positive correlates, whereas female sex and longer daily sedentary time were identified as negative correlates. For HL, monthly income and regular exercise were identified as positive correlates.

**Conclusion:**

Using an exploratory and variable-screening framework, this study identified a small set of socio-ecological correlates associated with PA and HL among middle-aged and older Chinese adults. Given the cross-sectional design, convenience sampling, and predominantly urban and well-educated sample, the findings should be interpreted as descriptive and hypothesis-generating rather than causal. Future studies using longitudinal designs and more representative samples are needed to confirm these associations and clarify their underlying mechanisms.

## 1 Introduction

Physical activity reduces the risk of mood disorders, cardiovascular and metabolic diseases, several cancers, and all-cause mortality (1). However, physical inactivity remains prevalent worldwide (2, 3), partly driven by urbanization-related environmental and lifestyle changes (4). Middle-aged and older adults are particularly susceptible to the consequences of inactivity, as insufficient activity can accelerate sarcopenia, bone loss, falls, and premature mortality (5, 6). In rapidly aging China—the world’s most populous country (7, 8)—promoting active lifestyles in midlife and later life is therefore a major public health priority.

Health literacy is increasingly recognized as an upstream determinant of health and health behaviors. It refers to individuals’ ability to access, understand, appraise, and use health information and services to maintain and promote health for themselves and others (9). Health literacy is socially patterned, being closely related to education, income, and other social determinants, and it influences disease prevention and adoption of health-promoting behaviors (10–13). For physical activity, higher health literacy may facilitate understanding of activity guidelines, identification of reliable information, and translation of recommendations into sustained behavioral routines through motivational, cognitive, and self-management processes. Although findings vary across settings and measures, existing evidence generally supports a positive association between health literacy and physical activity (14–16).

In China, marked urban–rural and regional disparities in living environments, socioeconomic resources, and access to health services may contribute to heterogeneity in both health literacy and physical activity. Middle-aged and older adults may experience cumulative disadvantages in education, income, and information access while managing functional decline and chronic disease, making these disparities particularly salient. Despite growing interest in both physical activity and health literacy, most studies have examined these constructs in isolation (17, 18), and fewer have integrated predictors across multiple socio-ecological levels—spanning individual characteristics, interpersonal relationships, and broader community/socioeconomic contexts—among middle-aged and older Chinese adults.

Guided by socio-ecological models, the present study aimed to identify multi-level factors associated with physical activity and health literacy among Chinese adults aged ≥40 years. We applied LASSO regression as a robust variable-screening approach to handle a broad set of socio-demographic, behavioral, interpersonal, and community/socioeconomic correlates. By clarifying socio-ecological correlates of both outcomes, this study seeks to inform the development of more targeted and context-sensitive strategies to promote active aging and strengthen health management capacity in China.

## 2 Methods

### 2.1 Participants and procedure

We collaborated with university teachers in several Chinese cities to recruit community-dwelling adults aged ≥40 years. Participants were initially invited through local networks and then asked to share the survey link within their social circles (snowball sampling). Convenience sampling was chosen to enable rapid data collection under limited resources.

The online questionnaire was administered between July and September 2023. Before entering the survey, participants read an information sheet and provided electronic informed consent. To ensure data quality, each device/IP address could submit only one completed questionnaire. Completion times were monitored, and records showing automated response patterns were removed. All data were processed confidentially and in line with relevant ethical and data protection standards.

Inclusion criteria were: (1) age ≥40 years (based on commonly used thresholds for middle-aged and older adults in China (19–21)); (2) voluntary participation and online consent; and (3) permanent residence in communities within official administrative boundaries. Exclusion criteria were: (1) completion time <270 s (determined via pilot testing as the minimum reasonable duration); and (2) duplicate or obviously invalid responses. After applying these criteria, 508 participants were retained for analysis.

### 2.2 Measures

Guided by socio-ecological frameworks and previous work (22–24), we grouped independent variables into four domains: individual characteristics, behaviors, interpersonal relationships, and community/socioeconomic factors.

#### 2.2.1 Individual characteristics

Age was grouped into six categories (40–44, 45–49, 50–54, 55–59, 60–64, ≥65 years). Body mass index (BMI) was calculated from self-reported height and weight. Educational attainment ranged from primary school or below to master’s degree or above (six levels). Biological sex was coded as male = 0, female = 1.

#### 2.2.2 Behaviors

Smoking, alcohol consumption, regular exercise (defined as engaging in physical activity according to a fixed temporal schedule, such as exercising at regular intervals or on specific days each week), presence of chronic disease, and regular health check-ups (defined consistently with regular exercise) were coded as binary variables (1 = yes, 0 = no). Sleep duration and daily sedentary time were each categorized into four levels (<5, 5–<6, 6–<7, and ≥7 h/day for sleep; <4, 4–<6, 6–<8, and ≥8 h/day for sedentary time). Notably, although the dependent variable in this study captured overall physical activity level, regular exercise was included as an independent variable. This is because regular exercise primarily reflects behavioral stability (e.g., consistency in adhering to planned activity versus frequently postponing or altering plans) rather than the total volume of physical activity per se, which is more directly determined by other dimensions such as activity frequency, intensity, and duration. This distinction is analogous to that in sleep research between sleep regularity and total sleep duration, which represent conceptually distinct constructs and have differential implications for health (25, 26).

#### 2.2.3 Interpersonal relationships

Marital status (single, married, divorced, widowed) and living arrangement (living alone vs. not) were assessed.

#### 2.2.4 Community and socioeconomic characteristics

Residential area was coded as urban vs. rural. Employment status included student, full-time employment, part-time employment, unemployed, retired, and farmer. Monthly personal income was grouped into five levels (≤3,000 to >12,000 RMB). Main source of income was classified as salary/wages, parental support, pension/retirement benefits, support from children/relatives, government subsidies, or other.

#### 2.2.5 Physical activity

Physical activity was assessed using the Physical Activity Rating Scale-3 (PARS-3) (27, 28). The scale contains three single items on exercise intensity, duration, and frequency, each rated on a 5-point scale. The total score is calculated as: Total score = intensity × (duration − 1) × frequency, with higher scores indicating greater physical activity.

#### 2.2.6 Health literacy

Health literacy was measured using the Chinese version of the 12-item Health Literacy Scale Short-Form (HLS-SF12) (27, 28). Items cover health care, disease prevention, and health promotion and are rated from 1 (very difficult) to 4 (very easy). The index is computed as: Index = (mean − 1) × (50/3), yielding scores from 0 to 50, with higher values indicating better health literacy. The HLS-SF12 has been widely used in Chinese populations (29–31).

### 2.3 Statistical analysis

Because we had many independent variables relative to the sample size, we first used LASSO regression to identify variables showing robust associations with each outcome (32–34). LASSO adds an L1 penalty term *λ* to the loss function, shrinking some coefficients to zero and yielding a parsimonious set of independent variables, which is generally more robust than stepwise selection (35). Continuous outcome data were modeled using LASSO-regularized linear regression under a Gaussian family (family = “gaussian”). Unordered categorical variables (e.g., marital status, primary economic source, and employment status) were coded as factors. Before applying LASSO, we calculated variance inflation factors (VIFs) to assess multicollinearity, using VIF > 5 as a threshold (36). According to this criterion, no evidence of multicollinearity was observed (Supplementary Table S1).

#### 2.3.1 Primary analysis: LASSO variable selection

We fitted LASSO regression models with an L1 penalty (alpha = 1) using glmnet, with variable standardization enabled (37). The regularization parameter (*λ*) was tuned via 10-fold cross-validation using cv.glmnet (nfolds = 10), with mean squared error as the evaluation metric (type.measure = “mse”). Two conventional tuning values were extracted: the λ yielding the minimum cross-validated error (λ_min) and the more parsimonious one-standard-error solution (λ_1se). Consistent with the goal of identifying a stable and interpretable factor set, the primary set of selected variables was defined as those with non-zero coefficients at λ_1se (excluding the intercept) (33). Penalized coefficients from this solution were reported descriptively and ranked by absolute magnitude. The minimal out-of-sample performance of the modeling procedure was quantified using cross-validated mean squared error (CV-MSE), root mean squared error (CV-RMSE), and cross-validated *R*^2^ based on held-out predictions from an outer cross-validation loop, defined as the proportional reduction in squared error relative to an intercept-only baseline.

#### 2.3.2 Sensitivity analysis 1: bootstrap stability of variable selection

To evaluate the robustness of LASSO-based selection to sampling variability, we conducted bootstrap resampling with replacement. Specifically, we generated bootstrap samples (1,000 times), with a size equal to the original sample size (38–40). Within each bootstrap sample, *λ* was tuned using 10-fold cross-validation under the same modeling settings. For each bootstrap replicate, we recorded whether each variable had a non-zero coefficient (selected vs. not selected) and the sign of the coefficient when selected. Across bootstrap replicates, we summarized (i) selection frequency (the proportion of replicates in which the coefficient was non-zero) and (ii) sign consistency (the proportion of positive vs. negative signs among replicates where the variable was selected). For interpretive purposes, variables meeting both a high-frequency and high-consistency criterion (selection frequency ≥0.70 and sign consistency ≥0.90) were flagged as stable signals (41–44). Median penalized coefficients across bootstrap replicates were additionally summarized as descriptive (shrunken) effect indicators.

#### 2.3.3 Sensitivity analysis 2: repeated random 10-fold cross-validation

As a complementary robustness check, we repeated the 10-fold cross-validation procedure 1,000 times using different random fold allocations in each repetition. In every repetition, LASSO models were tuned using cv.glmnet with identical settings, and the variable set was defined at λ_1se. Selection frequency and sign consistency were then computed across repetitions analogously to the bootstrap analysis.

#### 2.3.4 Sensitivity analysis 3: reanalysis excluding regular exercise

To avoid potential conceptual overlap between regular exercise and physical activity, we removed the regular exercise variable from the dataset and reran the analyses described above to examine whether the main associated factors and their selection patterns changed.

All stochastic components (bootstrap resampling and fold generation) were controlled using a fixed random seed (set.seed(20251115)), ensuring full reproducibility of the results.

## 3 Results

### 3.1 Participant characteristics

Among the 508 participants aged ≥40 years, 60.8% were men and 39.2% women (Table 1). Most were between 40 and 54 years old (70.6%), lived in urban areas (94.7%), and had at least a bachelor’s degree (85.3%). Regarding employment, 74.2% were full-time workers and 19.5% were retired; only 1.6% were farmers. The majority were married (92.3%) and not living alone (94.9%). Salaries were the main income source for 80.1% of participants, and 59.1% had monthly incomes between 5,001 and 12,000 RMB. With respect to behaviors, 27.0% smoked, 46.5% drank alcohol, 63.2% reported regular exercise, and 78.3% underwent regular health check-ups. Sedentary time was most commonly 4–<6 h/day (40.4%). Sleep duration typically ranged from 6–<7 h/day (45.3%) or ≥7 h/day (36.4%). A total of 29.5% reported at least one chronic disease. The mean (SD) BMI was 23.94 (2.81). Mean (SD) physical activity and health literacy scores were 18.45 (14.50) and 35.86 (7.93), respectively. Please note that because only one participant in the sample reported “student” as their employment status, this category was combined with the similarly small “farmer” group (*n* = 8). The merged category was subsequently labeled as “Other” (*n* = 9) in all subsequent analyses.

**Table 1:** Socio-demographic and health-related characteristics of participants (*N* = 508).

Variable	Category	*n* (%)	Mean (SD)	Median (IQR)
Sex	Male	309 (60.8)		
	Female	199 (39.2)		
Age group	40–44 years	139 (27.4)		
	45–49 years	108 (21.3)		
	50–54 years	111 (21.9)		
	55–59 years	61 (12.0)		
	60–64 years	51 (10.0)		
	≥65 years	38 (7.5)		
Residential area	Rural	27 (5.3)		
	Urban	481 (94.7)		
Education	1. Primary school or below	7 (1.4)		
	2. Junior high school	9 (1.8)		
	3. Senior high school/vocational school	19 (3.7)		
	4. Junior college	40 (7.9)		
	5. Bachelor’s degree	265 (52.2)		
	6. Master’s degree or above	168 (33.1)		
Employment status	1. Student	1 (0.2)		
	2. Full-time employment	377 (74.2)		
	3. Part-time employment	12 (2.4)		
	4. Unemployed	11 (2.2)		
	5. Retired	99 (19.5)		
	6. Farmer	8 (1.6)		
Marital status	1. Single	6 (1.2)		
	2. Married	469 (92.3)		
	3. Divorced	24 (4.7)		
	4. Widowed	9 (1.8)		
Living arrangement	5. Not living alone	482 (94.9)		
	6. Living alone	26 (5.1)		
Main source of income	1. Salary/wages	407 (80.1)		
	2. Pension/retirement benefits	76 (15.0)		
	3. Support from children/relatives	1 (0.2)		
	4. Government subsidies	1 (0.2)		
	5. Other	23 (4.5)		
Monthly income (RMB)	≤3,000	18 (3.5)		
	3,001–5,000	45 (8.9)		
	5,001–8,000	139 (27.4)		
	8,001–12,000	161 (31.7)		
	>12,000	145 (28.5)		
Smoking	No	371 (73.0)		
	Yes	137 (27.0)		
Drinking	No	272 (53.5)		
	Yes	236 (46.5)		
Regular exercise	No	187 (36.8)		
	Yes	321 (63.2)		
Regular check-ups	No	110 (21.7)		
	Yes	398 (78.3)		
Daily sedentary time	<4 h/day	157 (30.9)		
	4–<6 h/day	205 (40.4)		
	6–<8 h/day	105 (20.7)		
	≥8 h/day	41 (8.1)		
Sleep duration	<5 h/day	8 (1.6)		
	5–<6 h/day	85 (16.7)		
	6–<7 h/day	230 (45.3)		
	≥7 h/day	185 (36.4)		
Chronic disease	No	358 (70.5)		
	Yes	150 (29.5)		
BMI	–	–	23.94 (2.81)	23.81 (3.86)
Physical activity score	–	–	18.45 (14.50)	16.00 (18.25)
Health literacy index	–	–	35.86 (7.93)	33.33 (8.34)

### 3.2 LASSO-selected correlates of physical activity and health literacy

**Figure 1:**
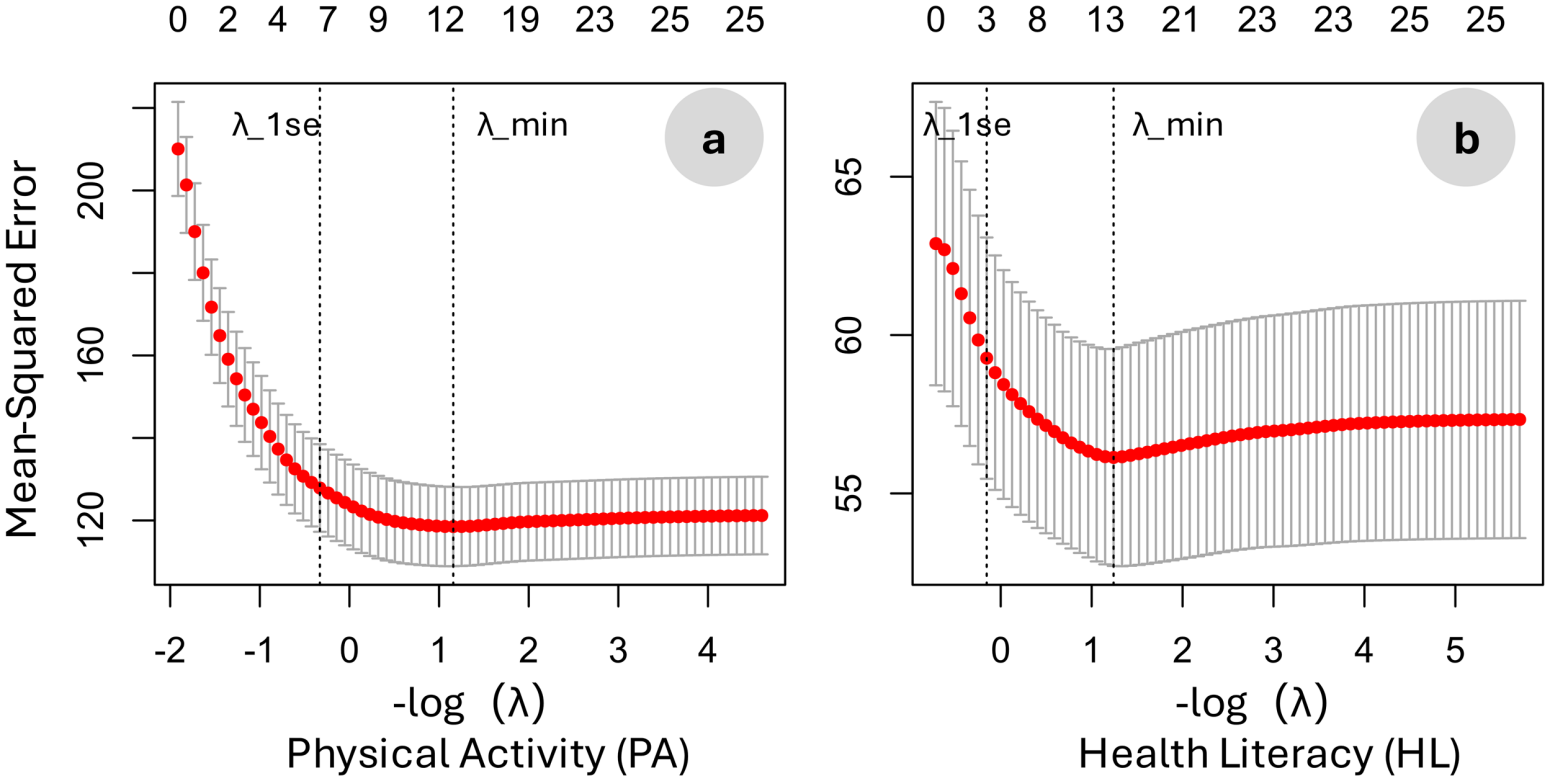
Relationship between mean squared error (MSE) and the penalty parameter (−log (*λ*)) in the LASSO regression: **(a)** physical activity as the outcome and **(b)** health literacy as the outcome.

Using LASSO regression with 10-fold cross-validation, correlates of physical activity (PA) and health literacy (HL) were identified separately (Table 2; Figure 1). For PA, the optimal penalization parameters were λ_min = 0.3140 and λ_1se = 1.3913, whereas for HL they were λ_min = 0.2890 and λ_1se = 1.1669. Results below focus on the more conservative λ_1se solutions. For physical activity, six correlates were observed, including regular exercise and sleep duration, sex and daily sedentary time, as well as two employment-status contrasts with opposite directions. For health literacy, only three correlates were observed: monthly income, regular exercise, and other employment status (full-time employment as the reference). Please note that “other employment status” does not represent a clearly defined category and therefore does not allow for meaningful interpretation. Therefore, this result is not reported or discussed further.

**Table 2:** Non-zero coefficients from LASSO models.

Correlates	Physical activity *β*	Health literacy *β*
Regular exercise (reference = no regular exercise)	9.66	0.61
Sleep duration	5.90	–
Sex (reference = male)	−2.21	–
Daily sedentary time	−1.33	–
Retired (reference = full-time employment)	0.55	–
Other employment status (reference = full-time employment)	−0.71	−5.14
Monthly income	—	0.61

Coefficients reported in the table are shrinkage estimates obtained under penalization; λ_1se was selected for the regression.

For physical activity, the λ_1se model achieved CV-MSE = 129.92 and CV-RMSE = 11.40, corresponding to a cross-validated *R*^2^ of 0.383. For health literacy, the corresponding performance was more modest (CV-MSE = 59.64; CV-RMSE = 7.72; CV-*R*^2^ = 0.053). Across both outcomes, λ_min solutions yielded slightly improved predictive performance but at the cost of reduced parsimony (Supplementary Table S2).

### 3.3 Resampling-based stability

For physical activity, four correlates consistently met predefined stability criteria (selection frequency ≥ 0.70 and sign consistency ≥ 0.90): regular exercise, sleep duration, sex, and daily sedentary time. For health literacy, three correlates met the same criteria: monthly income, regular exercise, and other employment status (with full-time employment as the reference) (Table 3). Repeated CV results closely mirrored bootstrap findings for both outcomes (Supplementary Table S3).

**Table 3:** Bootstrap stability.

Correlates	Outcome	Selection frequency	Sign consistency	Median *β*
Regular exercise (reference = no regular exercise)	PA	1.000	+ (100%)	9.407
Sleep duration	PA	1.000	+ (100%)	5.855
Sex (reference = male)	PA	0.981	− (100%)	−2.121
Daily sedentary time	PA	0.972	− (100%)	−1.320
Monthly income	HL	0.929	+ (100%)	0.675
Regular exercise (reference = no regular exercise)	HL	0.842	+ (100%)	0.934
Other employment status (reference = full-time employment)	HL	0.834	− (100%)	−4.734

Coefficients reported in the table are shrinkage estimates obtained under penalization. Only variables meeting the predefined criteria of selection frequency ≥0.70 and sign consistency ≥0.90 were considered eligible and are presented; Based on 1,000 resampling iterations and using λ1se for model estimation.

### 3.4 Re-analyses excluding regular exercise

For physical activity, five correlates were retained across all selection procedures: sleep duration (positive), routine health check-ups (positive), retired (with full-time employment as the reference) (positive), sex (negative), and daily sedentary time (negative). These variables were retained at λ_1se and showed high selection frequencies with consistent directions in both bootstrap resampling and repeated cross-validation (Supplementary Tables S4–S7). For health literacy, monthly income (positive) and other employment status (full-time employment as the reference) (negative) were the only correlates that consistently passed all procedures (Supplementary Tables S8–S11).

## 4 Discussion

### 4.1 Summary of main findings

Across both outcomes, LASSO with selection tests identified a small set of associated factors. For physical activity (PA), regular exercise and sleep duration were positively associated with PA, whereas sex (female vs. male) and daily sedentary time were negatively associated. For health literacy (HL), monthly income and regular exercise showed positive associations.

While the LASSO-selected correlates of physical activity showed moderate cross-validated explanatory power, the corresponding cross-validated *R*^2^ values for health literacy were small. The results suggest that, while several sociodemographic and behavioral correlates were robustly identified, a large proportion of variance in health literacy remains unexplained by the current set of correlates. This may be because the conceptualization of health literacy is a multifaceted construct shaped by long-term educational, cognitive, and contextual factors that are not fully captured by cross-sectional sociodemographic measures. In this context, the present findings are best interpreted as identifying correlates rather than providing a highly predictive model of health literacy.

### 4.2 Comparison with previous studies

Our finding that men have higher physical activity levels than women aligns with studies showing sex differences across multiple domains of physical activity (45) and lower prevalence of physical inactivity in retired Chinese men compared with women (17). These patterns likely reflect both biological factors and gendered divisions of labor and caregiving.

Regular exercise emerged as a correlate of overall physical activity in this study. In theory, exercise is only a subset of physical activity (46). Importantly, regular exercise was operationalized as a behavioral pattern characterized by temporal regularity—namely, the habitual engagement in planned or structured exercise over time—typically regarded as the opposite of irregular exercise (47). In contrast, overall physical activity captured a broader construct reflecting cumulative activity across daily life, including occupational, transport-related, and leisure-time domains (48). In our questionnaire, physical activity was assessed across three dimensions—frequency, typical duration, and intensity of activities of various types. In contrast, regular exercise specifically referred to whether individuals engaged in deliberate exercise according to a fixed schedule or at regular time points. The observed phenomenon may be attributable to that individuals who maintain regular exercise habits may develop stronger self-regulatory capacities, such as routine formation, planning, and behavioral consistency, which facilitate higher levels of physical activity beyond formal exercise sessions and across multiple contexts.

Our results also support prior evidence linking sleep and physical activity. Longer sleep duration has been associated with sufficient physical activity among older adults in institutional settings (49), although causal pathways remain unclear. The associations between higher physical activity, and less sedentary time in our study are consistent with the idea that these behaviors cluster and compete for limited daily time.

With respect to health literacy, our findings are broadly consistent with studies showing strong “social gradient” effects: higher socioeconomic status, including education status and income, is associated with better health literacy or health information literacy (50–52). We also observed robust associations between personal income and health literacy. Differences in conceptualization (health literacy vs. health information literacy), measurement (continuous vs. categorical outcomes), and sample characteristics (hospitalized vs. community-dwelling, older vs. middle-aged and older adults) may explain why education did not emerge as a major associated factor in our models.

Finally, it should be noted that in the sensitivity analyses excluding regular exercise, being retired (with full-time employment as the reference category) and regular health check-ups were identified as associated factors. Retirement is often accompanied by greater discretionary time and fewer occupational constraints, which may facilitate engagement in various forms of non-exercise physical activity, such as walking, household tasks, and leisure-time movement. In contrast, full-time employment may involve prolonged sedentary periods and limit opportunities for spontaneous or accumulated physical activity throughout the day. In addition, regular health check-ups may be associated with higher physical activity levels, as individuals who engage in preventive health services are generally more health-conscious and proactive in managing their health, which may extend to maintaining more active lifestyles.

### 4.3 Limitations

Several limitations should be considered. First, our convenience online snowball sampling likely selected individuals who are urban, digitally connected, and relatively well-educated, which limits generalizability to the broader population of middle-aged and older Chinese adults. Estimates for these subgroups are therefore likely unstable and should be interpreted with caution. Future research should use probability-based or stratified sampling, or deliberately oversample rural residents and farmers, to validate whether the observed socioeconomic and occupational patterns hold in more representative samples. Second, the cross-sectional design and reliance on self-reported measures for health literacy, physical activity, and behaviors may introduce recall and social desirability biases and do not permit causal inference. Third, we used short-form instruments, which cannot differentiate specific domains of physical activity or sub-dimensions of health literacy in detail. Finally, although we used LASSO to handle many variables with a modest sample size, results may still be sensitive to model specification and unmeasured confounding. Our findings should therefore be interpreted as preliminary and hypothesis-generating.

## 5 Conclusion

This cross-sectional, exploratory study examined socio-ecological correlates of physical activity and health literacy among middle-aged and older Chinese adults. A limited number of factors were identified as being associated with physical activity, including regular exercise patterns, sleep duration, sex, and daily sedentary time. For health literacy, monthly income and regular exercise were identified as correlates, although the overall explanatory power of the models for health literacy was modest. Overall, this study provides an initial, methodologically cautious overview of potential socio-ecological correlates of physical activity and health literacy in midlife and later adulthood in China. Future research employing longitudinal or experimental designs, richer contextual measures, and more representative samples is required to validate these associations and to better understand the complex pathways linking social context, health literacy, and physical activity across the life course.

## Data Availability

The raw data supporting the conclusions of this article will be made available by the authors, without undue reservation.
