# Supplementary material for: Socio-ecological factors of health literacy and physical activity among middle-aged and older Chinese adults
Source: Front Public Health. 2026 Feb 19;14:1749276. doi: 10.3389/fpubh.2026.1749276 (PMC12960079; doi:10.3389/fpubh.2026.1749276)
Supplement: Supplementary file 1 [file Table_1.DOCX]

Supplementary Material

**Table S1. The VIFs for independent variables**

| **Variable** | **Tolerance** | **VIF** |
| --- | --- | --- |
| Sex | 0.655 | 1.527 |
| Age group | 0.867 | 1.154 |
| Urban–rural residence | 0.929 | 1.077 |
| Monthly income | 0.854 | 1.172 |
| Smoking | 0.778 | 1.285 |
| Regular exercise | 0.826 | 1.211 |
| Regular health check-ups | 0.868 | 1.152 |
| Daily sedentary time | 0.911 | 1.098 |
| Sleep duration | 0.934 | 1.071 |
| Presence of chronic disease | 0.898 | 1.114 |
| BMI | 0.803 | 1.245 |

*Note:* only the variables treated as continuous variables are included in this test

**Table S2. Minimal out-of-sample performance (outer 10-fold CV)**

| Outcome | Solution | CV-MSE | CV-RMSE | CV-R² |
| --- | --- | --- | --- | --- |
| Physical activity | λ_1se | 129.92 | 11.40 | 0.383 |
|  | λ_min | 118.65 | 10.89 | 0.436 |
| Health literacy | λ_1se | 59.64 | 7.72 | 0.053 |
|  | λ_min | 56.33 | 7.51 | 0.105 |

**Note:** CV-R² represents the relative reduction in squared error compared with an intercept-only baseline.

**Table S3. Repeated CV stability**

| **Predictor** | **Outcome** | **Selection frequency** | **Direction (frequency)** |
| --- | --- | --- | --- |
| Regular exercise (yes vs. no) | PA | 1.000 | Positive (100%) |
| Sleep duration | PA | 1.000 | Positive (100%) |
| Sex (female vs. male) | PA | 1.000 | Negative (100%) |
| Daily sedentary time | PA | 1.000 | Negative (100%) |
| Monthly income | HL | 0.997 | Positive (100%) |
| Other employment status  (with full-time employment as the reference) | HL | 0.997 | Negative (100%) |
| Regular exercise (yes vs. no) | HL | 0.954 | Positive (100%) |

**Note:** Coefficients reported in the table are shrinkage estimates obtained under penalization. Only variables meeting the predefined criteria of selection frequency ≥ 0.70 and sign consistency ≥ 0.90 were considered eligible and are presented; Based on 1,000 repetitions and using λ1se for model estimation.

**Table S4. Non-zero coefficients from the LASSO model *(Physical activity outcome; regular exercise excluded)***

| **Predictor** | **β (λ₁se)** | **Direction** |
| --- | --- | --- |
| Sleep duration | 6.31 | Positive |
| Routine health check-ups (yes vs. no) | 3.11 | Positive |
| Retired (with full-time employment as the reference) | 2.46 | Positive |
| Sex (female vs. male) | −3.06 | Negative |
| Daily sedentary time | −2.43 | Negative |
| Other employment status (with full-time employment as the reference) | −2.98 | Negative |
| Chronic disease (yes vs. no) | −1.02 | Negative |

**Table S5. Minimal out-of-sample performance *(Physical activity outcome; regular exercise excluded)***

| **Solution** | **CV-MSE** | **CV-RMSE** | **CV-R² (vs intercept-only)** |
| --- | --- | --- | --- |
| λ₁se (primary) | 152.90 | 12.37 | 0.274 |
| λmin (optional) | 142.25 | 11.93 | 0.324 |

**Note:** CV-R² represents the relative reduction in squared error compared with an intercept-only baseline.

**Table S6. Bootstrap stability results *(Physical activity outcome; regular exercise excluded)***

| **Predictor** | **Selection frequency** | **Sign consistency** | **Median β** |
| --- | --- | --- | --- |
| Sleep duration | 1.000 | Positive (100%) | 6.162 |
| Daily sedentary time | 1.000 | Negative (100%) | −2.356 |
| Sex (female vs. male) | 0.990 | Negative (100%) | −2.774 |
| Routine health check-ups (yes vs. no) | 0.965 | Positive (100%) | 2.710 |
| Retired (with full-time employment as the reference) | 0.886 | Positive (100%) | 2.082 |

**Note:** Coefficients reported in the table are shrinkage estimates obtained under penalization. Only variables meeting the predefined criteria of selection frequency ≥ 0.70 and sign consistency ≥ 0.90 were considered eligible and are presented; Based on 1,000 resampling iterations and using λ₁se for model estimation.

**Table S7. Repeated cross-validation stability *(Physical activity outcome; regular exercise excluded)***

| **Predictor** | **Selection frequency** | **Direction** |
| --- | --- | --- |
| Sex (female vs. male) | 1.000 | Negative (100%) |
| Routine health check-ups (yes vs. no) | 1.000 | Positive (100%) |
| Daily sedentary time | 1.000 | Negative (100%) |
| Sleep duration | 1.000 | Positive (100%) |
| Retired (with full-time employment as the reference) | 0.999 | Positive (100%) |
| Other employment status (with full-time employment as the reference) | 0.787 | Negative (100%) |
| Chronic disease (yes vs. no) | 0.787 | Negative (100%) |

**Note:** Coefficients reported in the table are shrinkage estimates obtained under penalization. Only variables meeting the predefined criteria of selection frequency ≥ 0.70 and sign consistency ≥ 0.90 were considered eligible and are presented; Based on 1,000 repetitions and using λ1se for model estimation.

**Table S8. Non-zero coefficients from the LASSO model *(Health literacy outcome; regular exercise excluded)***

| **Predictor** | **β (λ₁se)** | **Direction** |
| --- | --- | --- |
| Other employment status (with full-time employment as the reference) | −4.62 | Negative |
| Monthly income | 0.54 | Positive |

**Table S9. Minimal out-of-sample performance *(Health literacy outcome; regular exercise excluded)***

| **Solution** | **CV-MSE** | **CV-RMSE** | **CV-R² (vs intercept-only)** |
| --- | --- | --- | --- |
| λ1se (primary) | 60.35 | 7.77 | 0.041 |
| λmin (optional) | 57.01 | 7.55 | 0.095 |

**Note:** CV-R² represents the relative reduction in squared error compared with an intercept-only baseline.

**Table S10. Bootstrap stability results *(Health literacy outcome; regular exercise excluded)***

| **Predictor** | **Selection frequency** | **Sign consistency** | **Median β** |
| --- | --- | --- | --- |
| Monthly income | 0.907 | Positive (100%) | 0.650 |
| Other employment status (with full-time employment as the reference) | 0.817 | Negative (100%) | −4.680 |

**Note:** Coefficients reported in the table are shrinkage estimates obtained under penalization. Only variables meeting the predefined criteria of selection frequency ≥ 0.70 and sign consistency ≥ 0.90 were considered eligible and are presented; Based on 1,000 resampling iterations and using λ1se for model estimation.

**Table S11. Repeated cross-validation stability *(Health literacy outcome; regular exercise excluded)***

| **Predictor** | **Selection frequency** | **Direction** |
| --- | --- | --- |
| Other employment status (with full-time employment as the reference) | 0.990 | Negative (100%) |
| Monthly income | 0.990 | Positive (100%) |
| Sleep duration | 0.086 | Positive (100%) |

**Note:** Coefficients reported in the table are shrinkage estimates obtained under penalization. Only variables meeting the predefined criteria of selection frequency ≥ 0.70 and sign consistency ≥ 0.90 were considered eligible and are presented; Based on 1,000 repetitions and using λ1se for model estimation.
